# Supplementary material for: Nutritional Status According to the GLIM Criteria in Patients with Chronic Heart Failure: Association with Prognosis
Source: Nutrients. 2022 May 27;14(11):2244. doi: 10.3390/nu14112244 (PMC9182728; doi:10.3390/nu14112244)
Supplement: Supplementary file 1 [file nutrients-14-02244-s001.zip › nutrients-1702100-supplementary.pdf]

nutrients

MDPI

Supplementary Materials

Nutritional Status According to the GLIM Criteria in Patients with Chronic Heart Failure: Association with Prognosis

Clara Joaquín [1,2,†,*], Nuria Alonso [1,2,†], Josep Lupón [2,3,4,5], Paloma Gastelurrutia [3,4], Alejandra Pérez-Monstesdeoca [1], Mar Domingo [5], Elisabet Zamora [2,4,5], Guillem Socias [1], Analía Ramos [12], Antoni Bayes-Genis [2,3,4,5,‡] and Manel Puig-Domingo [1,2,‡]

Table S1. Competing risk strategy by Gray method results for factors potentially related to the 5-year CV mortality, considering non-cardiovascular mortality as the competing event for cardiovascular death.

Factor	5-year CV mortality					
	Univariate		Multivariate GLIM		Multivariate MNA	
	HR (95% CI)	*p*-value	HR (95% CI)	*p*-value	HR (95% CI)	*p*-value
Age	**1.15 (1.11–1.19)**	**< 0.001**	**1.11 (1.07–1.15)**	**< 0.001**	**1.13 (1.09–1.18)**	**< 0.001**
Sex	**6.35 (3.08–13.07)**	**< 0.001**	---	---	---	---
BMI	**1.11 (1.03–1.20)**	**0.004**	**---**	**---**	**1.09 (1.01–1.17)**	**0.015**
NYHA class	**96.17 (43.6-211.7)**	**< 0.001**	**34.19 (13.25–88.21)**	**< 0.001**	**22.90 (9.36–56.01)**	**< 0.001**
MNA[a]	**14.82 (6.63–33.12)**	**< 0.001**	NE	NE	**10.19 4.54–22.88**	**< 0.001**
GLIM criteria[a]	2.16 (0.97–4.83)	0.06	---	---	NE	NE
Barthel index	**0.93 (0.91–0.95)**	**< 0.001**	**---**	**---**	---	---
Diabetes Mellitus	**6.31 (3.22–12.37)**	**< 0.001**	**2.30 (1.15–4.61)**	**0.018**	**2.12 (1.09–4.14)**	**0.027**

Bold values indicate factors significantly related to mortality. [a] For analyses MNA and GLIM criteria categories were merged in normal nutritional status and abnormal nutritional status. BMI = Body Mass Index. NYHA = New York Heart Association; MNA = Mini Nutritional Assessment; MN = Malnutrition; P-values were based on Cox regression analysis. NE = Non-evaluated.

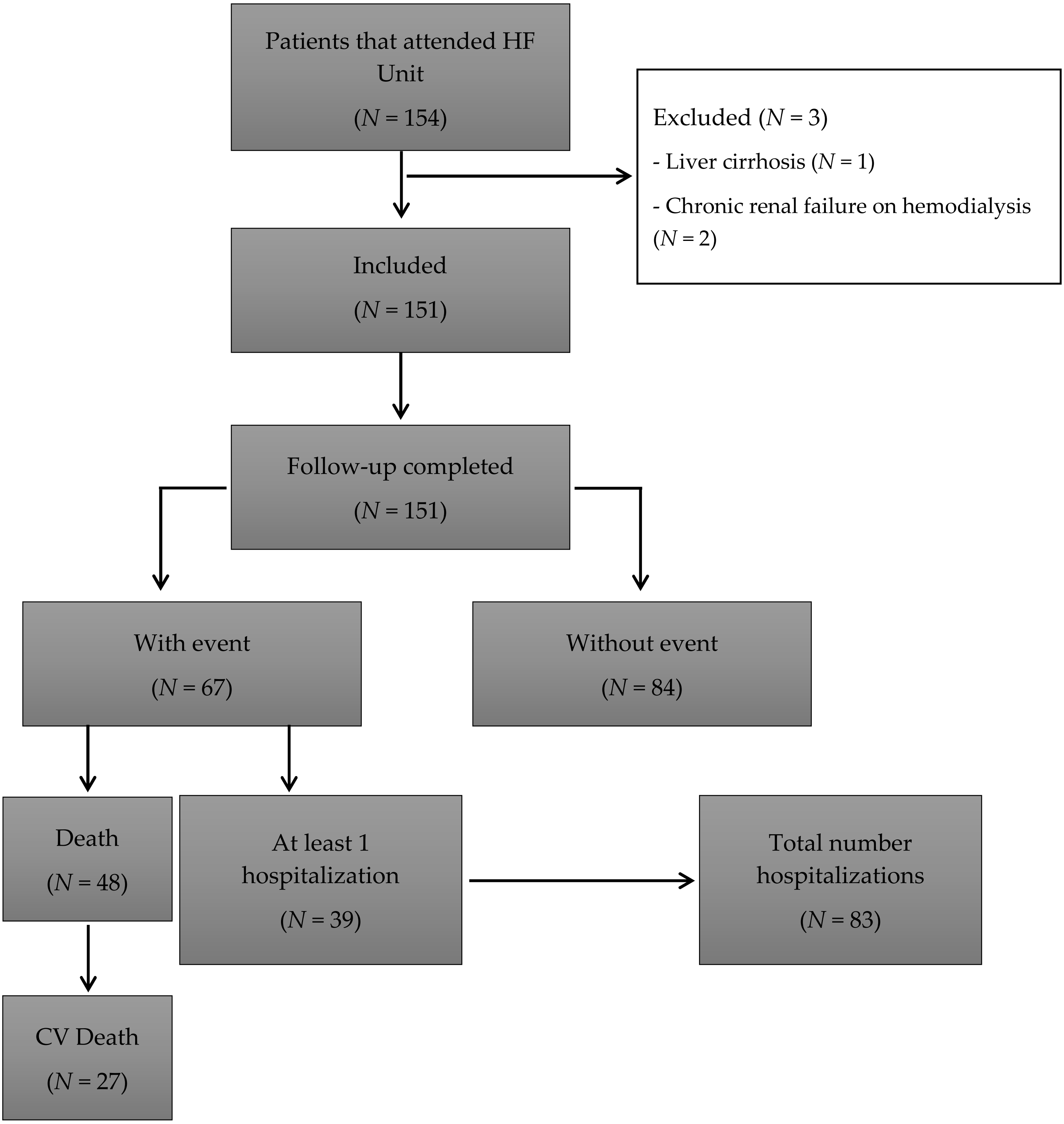

Figure S1. Study flowchart.
